# A Bizarre Presentation of Osteoid Osteoma of Maxilla

**Published:** 2016-10-01

**Authors:** Tanya Khaitan, Pachigolla Ramaswamy, Uday Ginjupally, Arpita Kabiraj

**Affiliations:** 1 *Dept. of Dentistry, * *Murshidabad Medical College and Hospital, Berhampore, West Bengal, India*; 2 *Dept. of Oral Medicine & Radiology, St. Joseph Dental College & Hospital, Eluru, Andhra Pradesh, India*; 3 *Dept. of Oral Medicine & Radiology, Kamineni Institute of Dental Sciences, Narketpally Andhra Pradesh, India*; 4 *Dept. of Oral Pathology & Microbiology, Index Institute of Dental Sciences, Indore, Madhya Pradesh, India*

**Keywords:** Bone, Genesis, Neoplasm, Nidus

## Abstract

Osteoid osteoma (OO) is a benign osteogenic lesion that is extremely rare in jaws. It is characterized by proliferation of either cancellous or compact bone and can be central, peripheral or extraskeletal. Pain is a distinctive feature of this lesion accompanied by vasomotor disturbances, which occur long before radiographic and histopathology findings manifest. Here, we present a rare case report of OO of maxilla in a 40-yr-old male patient with noteworthy clinical, radiological and histological presentation. The diagnosis of OO is usually obtained by radiographs confirmed by histopathological analysis. Thus, the oral physician should have keen observation and appropriate knowledge concerning the same to avoid confusion with similar bony lesions.

## Introduction

Osteoid osteoma (OO) is a benign tumor of bone accounting for less than 1% in jaws. It was first described as a distinctive clinical entity (1). It was referred as “sui genris”, amplifying the lesion’s small and self-limiting nature. Lichtenstein defined OO as “a small, oval, or roundish tumor-like nidus composed of osteoid and trabeculae of newly formed bone deposited within a substratum of highly vascularized osteogenic connective tissue” (2, 3).

This entity accounts for 3% of all primary bone tumors and 10% of benign bone tumors. PubMed Central literature review revealed only seven cases involving the jaws for the last 15 yrs (3). The exceptionality and rarity of this tumor make it enigmatically essential for the clinicians for a proper diagnosis and treatment. Thus, such case reports should be discussed to enlighten and strengthen our perception in the concerned field.

Here, we presented a rare case report of OO of maxilla in a 40-yr-old patient.

## Case report

A 40-yr-old male patient presented with swelling in the left upper front jaw region since 3 months. The swelling was gradual in onset and slowly increased in size to attain the present size. It was associated with mild intermittent pain, bleeding, excessive salivation and loosening of tooth. There was no history of paresthesia or numbness. Past medical, dental and personal histories were inconspicuous. No abnormality was noted on general physical and extraoral examination. Informed consent and ethical clearance were obtained from the patient.

**Fig. 1:**
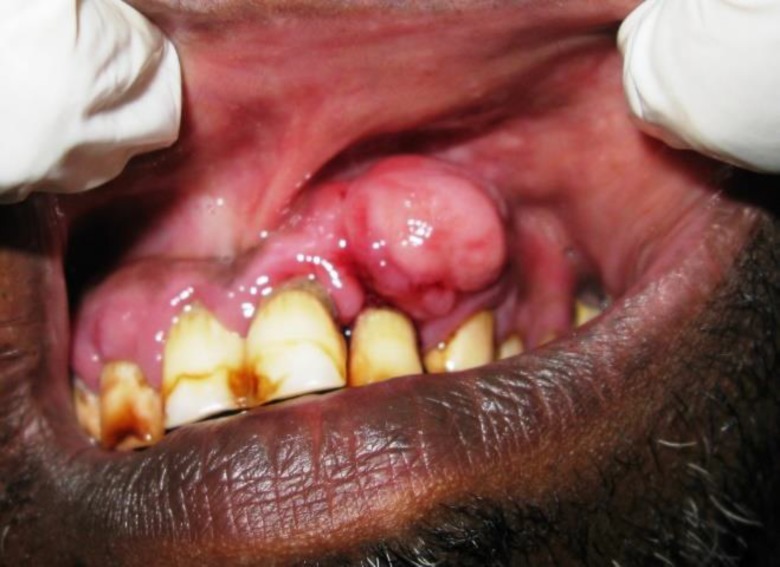
Solitary, well defined, dome-shaped, sessile swelling in the labial aspect of 22, 23 regions

Intraoral examination revealed presence of solitary, well-defined, dome-shaped swelling in the labial aspect of 22, 23 regions measuring approximately 2 x 1 cm in size. Surface over the swelling appeared lobulated and of the same color as the adjacent mucosa. On palpation, it was tender, sessile, firm to hard in consistency, and non-fluctuant. Bleeding was elicited on provocation. Hard tissue examination showed grade III mobility in relation to 22 (Fig. 1).

**Fig. 2:**
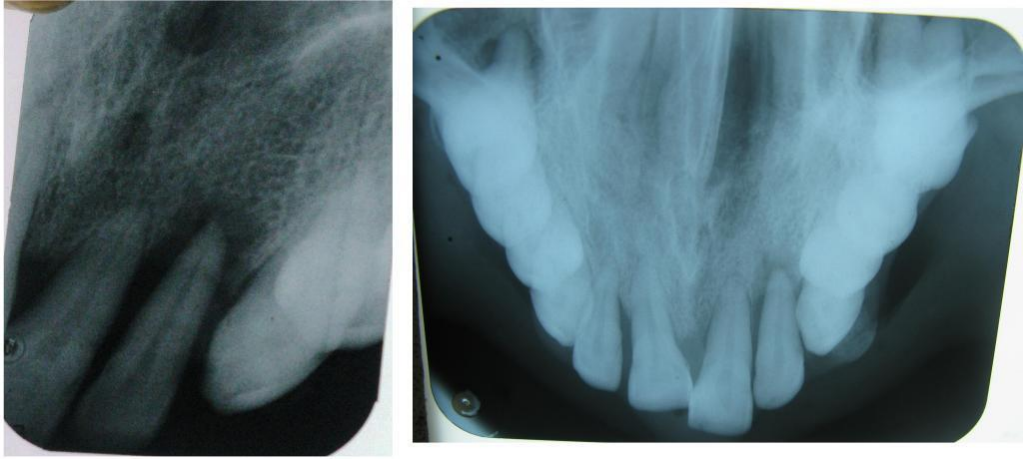
Radiographical representation (IOPAR and maxillary cross-sectional occlusal view

The patient was further subjected to radiological examination. Intraoral periapical radiograph of 22, 23 regions revealed ill-defined homogenous periarticular radiolucency in relation to 22. Maxillary cross-sectional occlusal radiograph showed well-defined radiopaque nidus surrounded by thin radiolucent border in 22, 23 regions (Fig. 2). Based on history, clinical and radiological examination, a differential diagnosis of ossifying fibroma, peripheral osteoma and osteoblastoma was considered.

**Fig. 3:**
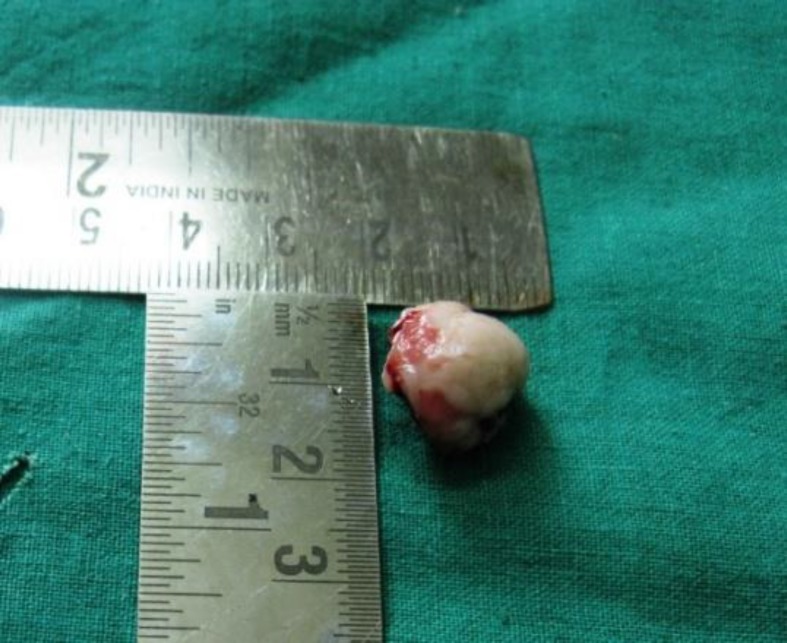
Gross tissue specimen of surgically excised tissue

**Fig. 4:**
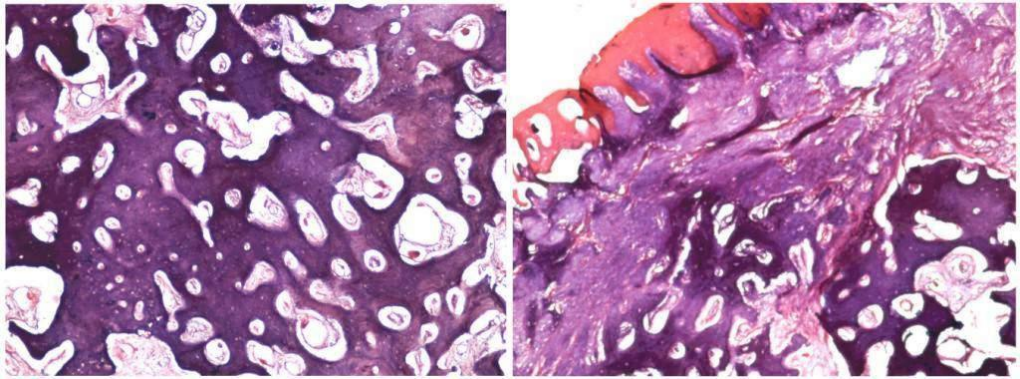
Photomicrograph of H & E stained section showed well-circumscribed lesion consisting of interconnected trabeculae of bone with loose vascular matrix composed of numerous large dilated capillaries. The center of the lesion composed of compact mineralized bone with presence of cemental lines. (X20 magnifications

The hematological examination was within satisfactory limits. The patient was subjected to excisional biopsy along with extraction of 22 and the specimen sent for histopathological analysis (Fig. 3). The photomicrograph showed well-circumscribed lesion lined by intact stratified squamous epithelium. The connective tissue stroma consisted of interconnected trabeculae of bone with loose vascular matrix composed of numerous large dilated capillaries. The center of the lesion was made up of compact mineralized bone with presence of cemental lines. There was no line of demarcation between the lesion and the surrounding tissue (Fig. 4). Based on all the above features, final diagnosis of osteoid osteoma was established.

The patient was kept under periodic follow-up for 1, 3 and 6 months. No recurrence was reported. 

## Discussion

OO is a benign tumor of the bone rarely involving the craniofacial bones. The precise nature of this lesion remains uncertain. Some authors consider it as low growth and inactive neoplasm or an inflammatory reaction or a consequence of an unusual healing process (3).

Jaffe (1935), had set certain criteria for OO: a) benign neoplasm; b) formed large amounts of osteoid which became calcified; c) an inflammatory process; d) characteristic radiographic changes, such as focal rarefaction and reactive bone formation; e) occurred most frequently in young adults; f) an outstanding feature of pain; and g) complete removal as the treatment of choice (4).

Various theories have been put forward regarding the nature and genesis of OO. Jaffe (1935) considered it as a variant of osteoblastoma (4). In the latest stages of development, OO was observed apparent histological patterns of a pronounced neoplastic lesion (2). The lesion considered of embryonal nature and as a hamartoma. An inflammatory lesion portrayed as pain was its invariable feature (5). The cases involving the jaws were reviewed as a type of nidus, more brittle in nature, comprising of osteoid tissue predominantly. Lichtenstein correlated that microscopically, a broken nidus may be misguided as granulation tissue and the older lesion might show atypical bone modeled from sheets of osteoid trabeculae (6).

OO generally involves the tibia, femur, fibula, humerus and vertebral arch. Jaw involvement is rare, lingual surface and lower border of the body of mandible being the most common sites. It is seen more commonly in males compared to females with a ratio of 2:1, affecting in the second and third decades of life (6). This finding was consistent in the above case. In contrary, the present case was observed in the maxillary anterior region.

OO is clinically characterized by dull, throbbing, intermittent, local and nocturnal pain relieved with aspirin and associated with slight local swelling. Various theories were brought forward to elucidate the reason for pain. Arteriolar blood supply was observed to the lesion whereas others reported pressure exertion upon the surrounding bone, suggesting that the pain was produced by the vascular tumor lying within the confines of the sclerosed bone trabeculae (7).

The classical radiological appearance of OO is a small, radiolucent intracortical nidus, <1 cm in diameter, surrounded by a large, dense sclerotic zone of cortical thickening. Jaffe emphasized these features to be the definitive diagnosis of this lesion. The radiodensity of the nidus was considered to be less and surrounded by a reactive radiopacity at a variable distance from the same. Radiopaque nidus suggested a less mature lesion whereas radiolucent nidus was indicative of a fully mature OO. Advanced imaging modalities like computed tomography and scintigraphy are considered as a useful adjunctive test (2).

The histological appearance of OO varies with the age of the lesion and its site. Huvos distinguished three distinct evolutionary stages of modification of OO. Initially, dense osteoblasts are seen proliferating actively in a highly vascularized stroma followed by deposition of osteoid matrix between the osteoblasts in the intermediate phase. In the mature stage, the osteoid is transformed into well-calcified, compact trabeculae of atypical bone, which are neither typically woven nor lamellar (1).

Ossifying fibroma, peripheral osteoma, and osteoblastoma were considered as differential diagnosis for the aforementioned case. Ossifying fibroma and peripheral osteoma are usually asymptomatic, increases in size, lack nidus and cause resorption and displacement of teeth. Osteoblastoma and OO are clinical, radiographically and histologically very similar lesions. Although pain is a common presenting feature, pain associated with OO response to aspirin and other nonsteroidal anti-inflammatory drugs, whereas an osteoblastoma does not (8).

Complete excision is considered as the treatment of choice. The nidus should be removed intact. If the entire lesion is not removed or destroyed, the clinical complaints will remain and recur at a later stage. Malignant transformation of OO has not been reported in the literature until date (2, 6).

## Conclusion

The diagnosis of OO is usually obtained by radiographs and is confirmed by histopathological analysis. The oral physician should have keen observation and apt knowledge concerning the same to avoid confusion with similar bony lesions.

## Conflict of Interests

The authors declare that there is no Conflict of Interests. 
